# Eukaryotic Translation Elongation Factor 1-Alpha 1 Inhibits p53 and p73 Dependent Apoptosis and Chemotherapy Sensitivity

**DOI:** 10.1371/journal.pone.0066436

**Published:** 2013-06-14

**Authors:** Alvaro Blanch, Fiona Robinson, Ian R. Watson, Lynn S. Cheng, Meredith S. Irwin

**Affiliations:** 1 Department of Paediatrics and Cell Biology Program, Hospital for Sick Children, University of Toronto, Toronto, Ontario, Canada; 2 Departments of Medical Biophysics and Laboratory Medicine and Pathobiology, University of Toronto, Toronto, Ontario, Canada; German Cancer Research Center, Germany

## Abstract

The p53 family of transcription factors is a key regulator of cell proliferation and death. In this report we identify the eukaryotic translation elongation factor 1-alpha 1 (eEF1A1) to be a novel p53 and p73 interacting protein. Previous studies have demonstrated that eEF1A1 has translation-independent roles in cancer. We report that overexpression of eEF1A1 specifically inhibits p53-, p73- and chemotherapy-induced apoptosis resulting in chemoresistance. Short-interfering RNA-mediated silencing of eEF1A1 increases chemosensitivity in cell lines bearing wild type p53, but not in p53 null cells. Furthermore, silencing of eEF1A1 partially rescues the chemoresistance observed in response to p53 or p73 knockdown, suggesting that eEF1A1 is a negative regulator of the pro-apoptotic function of p53 and p73. Thus, in the context of p53-family signaling, eEF1A1 has anti-apoptotic properties. These findings identify a novel mechanism of regulation of the p53 family of proteins by eEF1A1 providing additional insight into potential targets to sensitize tumors to chemotherapy.

## Introduction

The p53-family proteins are transcription factors that play important roles in tumorigenesis through the regulation of genes involved in cell cycle progression, senescence and apoptosis. The three paralogues (p53 p63, and p73) share significant structural and functional similarity, including conserved transactivation (TA), DNA binding (DBD) and oligomerization (OD) domains. Due to alternative splicing and differential promoter usage, *p73* encodes protein isoforms that differ at the amino- (ΔN and TA) and carboxyl-termini (α, β, γ, etc) [1]. The ΔN isoforms lack the N-terminal transactivation domain present in the full-length transactivation competent (TA) isoforms. ΔN p73 and p63 proteins can act as dominant negative inhibitors of the pro-apototic full-length TAp73, TAp63 and p53 by forming inactive transcriptional tetramers [2], [3], [4].

Unlike p53, which is mutated or inactivated in more than 50% of human tumors [5], *p73* and *p63* mutations are rarely observed in cancers [6]. Instead high levels of ΔN p53 family proteins are commonly observed in human tumors and like p53, TAp73 is a tumor suppressor gene that when specifically deleted in mice (*TAp73-/-*) leads to enhanced tumor susceptibility. TAp73 also mediates chemotherapy-induced apoptosis, while ΔNp73 and ΔNp63 expression leads to chemoresistance [4], [7], [8]. These studies support a paradigm in which the balance between the various pro-apoptotic TA and anti-apoptotic ΔN p53 family isoforms determine whether specific p53 family-dependent signaling pathways lead to apoptosis or survival in tumor cells in response to specific stimuli including oncogenic stresses and DNA damage [9]. Significant research has identified proteins that interact with all p53 paralogs and their isoforms including a subset that selectively binds to and regulates only specific p53, p63 or p73 isoforms. To date, p53 family protein interacting partner proteins include kinases and ubiquitin ligases that mediate post-translational modifications and regulate stability (e.g. abl, HDM2, itch, Pirh2), co-activators and repressors that modulate transcriptional activities (e.g. PML, ASPPs), and proteins that regulate subcellular localization (e.g. Hipk2, MDMX) [10].

The eukaryotic translation elongation factor 1-alpha 1 (eEF1A1) is one of two isoforms of the alpha subunit of the elongation factor-1 complex (eEF1A1 and eEF1A2), which are encoded by genes that share 92% sequence identity (*EEF1A1*, NM_001402 and *EEF1A2*, NM_001958). The eEF1A1 isoform is ubiquitously expressed while eEF1A2 is detected in brain, heart and skeletal muscle. The eEF1A1 is a 50 KDa GTPase that couples the hydrolysis of GTP to GDP with the delivery of aminoacyl tRNAs to the ribosome during protein translation [11], [12]. eEF1A also has translation-independent roles in embryogenesis, senescence, oncogenic transformation, cell proliferation, apoptosis, cytoskeletal organization and protein degradation [12], [13], [14], [15], [16], [17], [18]. Both eEF1A1 and eEF1A2 have similar canonical translation elongation functions but may differ in their non-canonical functions.

Several lines of evidence support the importance of eEF1A1 in tumorigenesis. High levels of eEF1A1 have been reported in melanomas and tumors of the pancreas, breast, lung, prostate and colon [19], [20], [21], [22], [23], [24]. Increased eEF1A correlates with metastatic potential in mammary adenocarcinoma [25] and carcinogen-mediated transformation of fibroblasts [13]. The role of eEF1A in apoptosis is controversial and there are reports demonstrating both pro- and anti-apoptotic properties. The eEF1A is a p53 target gene [14] and several studies have reported increased expression of eEF1A in response to p53 activation, oxidative and endoplasmic reticulum stress-induced apoptosis [14], [26], [27], [28]. Moreover, some of these reports also demonstrated that inhibition of eEF1A protects against apoptosis [26], [27], [28] and ectopic overexpression of eEF1A induces apoptosis of fibroblasts [26]. In contrast, other studies have demonstrated anti-apoptotic properties of eEF1A1. High levels of eEF1A1 were associated with pro-survival activity and resistance to chemotherapy [24], [29] and down-regulation of eEF1A1 expression resulted in cell death [30]. Furthermore, ectopic eEF1A1 expression can protect cells from apoptosis [30], [31] while inhibition of eEF1A1 enhanced apoptosis [29], [30], [32], [33], [34] in a variety of cellular models. Some studies also suggest differential apoptotic effects for eEF1A1 and eEF1A2. In myotubules, apoptosis was rescued by overexpression of eEF1A2 or inhibition of eEF1A1, while overexpression of eEF1A1 accelerated apoptosis [35]. These reports demonstrating opposing properties of eEF1A1 suggest its effects may be cell context specific or may depend on interactions with different regulatory binding partners.

In this report we identify eEF1A1 to be a novel interacting partner of p53 and p73. eEF1A1 specifically inhibits p53-, TAp73- and chemotherapy-induced apoptosis. Interestingly, siRNA mediated knockdown of eEF1A1 increases chemotherapy-induced apoptosis in cell lines with wild-type p53, but not in cells lacking p53. Furthermore, silencing of eEF1A1 partially rescues the chemoresistance observed in response to p53 or TAp73 knockdown. These findings suggest that eEF1A1 is a negative regulator of the pro-apoptotic function of p53 and TAp73 and thus, in the context of p53-family signaling, eEF1A1 has anti-apoptotic properties.

## Materials and Methods

### Cell culture and drugs

Cervical carcinoma HeLa, osteosarcoma SaOS-2 and U2OS, human embryonic kidney HEK293A, and lung carcinoma A549 cells were obtained from American Type Culture Collection (Rockville, MD) and maintained in Dulbecco's Modified Eagle Medium (DMEM) (Gibco-Invitrogen, Gran Island, NY) supplemented with 10% heat-inactivated fetal bovine serum (FBS) (Hyclone, Logan, UT) at 37°C in a humidified 5% CO_2_ atmosphere. Colon carcinoma HCT116 *p53^+/+^* cells [36] were grown in McCoy's 5A medium (Gibco-Invitrogen). Osteosarcoma SaOS-2 cells stably transfected with the T7-p73DDα (carboxy-terminal region of p73α, amino acids 327–636) [37] were previously described [38]. Camptothecin, cisplatin, doxorubicin and etoposide (VP-16) (Sigma, St. Louis, MO) were dissolved according to manufacturer's instructions.

### Plasmids

pcDNA3-HA-TAp73α, pcDNA3-HA-ΔNp73α, pcDNA3-HA-p53, pcDNA-T7-p73DD were previously described [37].

Full-length eEF1A1 and eEF1A2 clones purchased from GeneCopoeia (Rockville, MD) and The Centre for Applied Genomics (Toronto, ON), respectively, were PCR amplified and subcloned into pcDNA3.1 vector (Invitogen) with the indicated amino terminal tags using the EcoRI and XhoI restriction sites.

### Silver stain and mass spectrometry

SaOS-2 cells transfected with a T7-p73DDα [37], [38] were treated overnight with camptothecin (0.2 µM) and nuclear fractions were immunoprecipitated with anti-p73 (ER-15, GC-15) or control antibodies. Immunoprecipitates were resolved on 10–15% SDS-PAGE gradient gels and then subjected to silver staining. Specific p73 immunoprecipitated bands were isolated from the silver stained gel, trypsinized and analyzed by mass spectrometry.

### Transfection and siRNA knockdown

Plasmids were transiently transfected into cells using either the PEI (polyethylenimine) method or FuGENE 6 (Promega, Madison, WI) according to manufacturer's instructions. siRNA transfection was performed as previously described [39]. Briefly, oligonucleotides (Dharmacon, Lafayatte, CO) at a final concentration of 75 nM were transfected with oligofectamine (Invitrogen) according to manufacturer's instructions. siRNA sequences for eEF1A1, p53 and TAp73 were previously described [39], [40], [41]. siGENOME RISC-Free Control siRNA (Dharmacon) was used as the negative control.

### Cell lysis, fractionation, immunoprecipitation and immunoblot

Immunoprecipitation and immunoblot procedures were performed as previously described [2], [39], [42]. Briefly, cells were lysed in EBC buffer (50 mM Tris pH 8, 120 mM NaCl, 0.5% Nonidet P-40) supplemented with complete protease inhibitors (Roche, Manheim, Germany). Equal amounts of whole cell extract as determined by Bradford method (Bio-Rad, Hercules, CA) were either resolved by SDS-PAGE or subjected to immunoprecipitation with the indicated antibodies and protein A-sepharose (Amersham Biosciences, Amersham, UK) for 2 h at 4°C. Immunoprecipitates were washed five times with NETN buffer (2 M Tris pH 8, 5 M NaCl, 0.5 M EDTA pH 8, 0.5% Nonidet P-40), eluted by boiling in SDS-containing sample buffer and resolved by SDS-PAGE. Proteins were transferred to nitrocellulose membrane (Bio-Rad) for western analysis with secondary antibodies conjugated with horseradish peroxidase (Pierce Rockford, IL) and detected by enhanced chemiluminescence SuperSignal kit (Pierce).

For nuclear fractionation cell pellets were incubated in buffer A (Tris-HCl 10 mM pH 7.6, KCl 75 mM, MgCl_2_ 5 mM, EDTA 1 mM, Triton X-100 0.5%) supplemented with DTT 1 mM, PMSF 1 mM and complete protease inhibitors (Roche). Following 5 min incubation on ice, the nuclear fraction was pelleted by centrifugation at 200 g for 10 min at 4°C, and the cytoplasmic fraction was decanted. Following wash with buffer A the nuclear pellet was lysed with buffer B (Tris-HCl 20 mM pH 7.6, KCl 50 mM, NaCl 400 mM, Triton X-100 1%, Glycerol 10%) supplemented with 1 mM DTT, 1 mM PMSF and complete protease inhibitors (Roche) and centrifuged for 10 min at 16000 g at 4°C.

### Antibodies

The following monoclonal antibodies were used: anti-eEF1α and anti-vinculin (Upstate-Millipore, Lake Placid, NY), anti-T7 (Novagen, Madison, WI), anti-p21 (Cell Signalling Technology, Beverly, MA), anti-p53 (DO-1) (Calbiochem, San Diego, CA), anti-HA (HA.11) (Covance, Denver, PA), and anti-Flag (Sigma-Aldrich, St. Louis, CA). Monoclonal antibodies anti-p73 ER-15 and GC-15 were previously described [2].

The following polyclonal antibodies were used: anti-GFP and anti-cleaved PARP (Upstate-Millipore, Lake Placid, NY), anti-HA (Y-11) (Santa Cruz Biotechnology, Santa Cruz, CA), and anti-p73 (BL906) (Bethyl Laboratories, Montgomery, TX).

### Caspase-3/7 assays

For caspase activation assays 24 hours after transfections cells were trypsinized, re-seeded in 96-well plates, and treated with the indicated chemotherapies. Caspase 3 and 7 enzymatic activity was determined using the Apo-ONE caspase-3/7 assay (Promega, Madison, WI) according to the manufacturer's instructions. Fluorescence was measured following 18 hours of substrate incubation in a Versa Max plate reader (Molecular Devices, Sunnyvale, CA). To control for differences in viability between study and control conditions, a parallel 96-well plate was seeded and the Cell Proliferation Kit I (MTT) (Roche, Manheim, Germany) was used according to manufacturer's instructions, reading absorbance at 562 nm with an ELx800 plate reader (Bio-Tek Instruments, Winooski, VT).

### RT-PCR

A total of 1 µg of RNA, isolated using the TRIzol reagent (Invitrogen Life Technologies), was used for reverse transcription with the Omniscript RT kit (Qiagen). The PCR amplification was performed using *p53* (forward: 5′ ttcctcttcctacagtactc, and reverse: 5′ gcaaatttccttcactcgg) and *GAPDH* (forward: 5′ gtggacctgacctgccgtct, and reverse: 5′ tagcccaggatgcccttgag) specific primers with an annealing temperature of 55°C.

### Cycloheximide assay

Cells grown in 6-well plates were transfected with siRNA oligonucleotides. Forty-eight hours after transfection cells were treated with 40 µg/mL cycloheximide (CHX) (Sigma, St Louis, MO) and lysed at the indicated times. Equal amounts of whole cell extracts were resolved by SDS-PAGE and immunoblotted with the indicated antibodies. IRDye-conjugated secondary antibodies and the Odyssey Infrared Imaging System (Li-Cor Biosciences, Lincoln, NE) were used for protein detection and quantification.

## Results

### eEF1A1 is a novel interacting partner of p53 and TAp73

p73 plays an important role in chemosensitivity [40]. In order to elucidate the mechanisms by which p73 induces apoptosis we sought to identify proteins that interact with p73 in response to DNA damaging chemotherapy agents. Nuclear extracts of SaOS-2 (*p53-/-*) osteosarcoma cells that stably express the carboxy-terminal fragment of p73α (p73DD) [37], [38] treated with camptothecin were immunoprecipitated either with anti-p73 antibodies or control antibodies. A 50 KDa protein that specifically co-immunoprecipitated with anti-p73, but not control antibodies, was identified by mass spectroscopy as eukaryotic translation elongation factor 1-alpha 1 (eEF1A1) (Data not shown).

**Figure 1:**
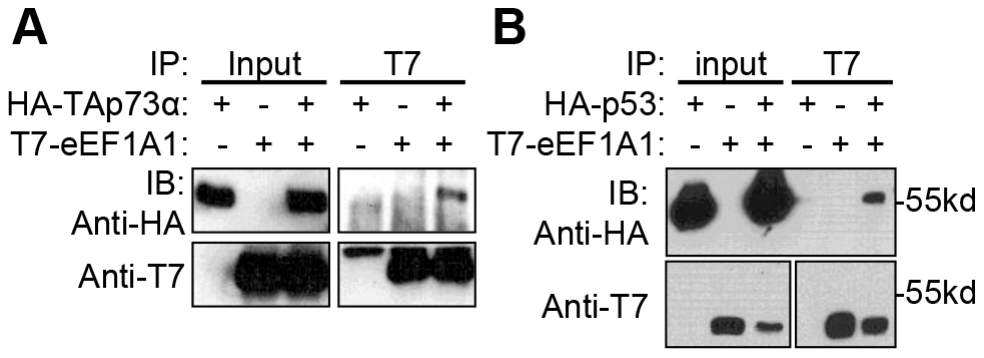
eEF1A1 interacts with p73 and p53. **Figure 1A**, HeLa cells were transfected with plasmids encoding HA-TAp73α alone or together with T7-eEF1A1. Equal amounts of whole cell extracts were immunoprecipitated with anti-T7 antibody, resolved by SDS-PAGE and immunoblotted with the indicated antibodies (anti-HA Y-11). **Figure 1B**, SaOS-2 cells were transfected with plasmids encoding HA-p53 and T7-eEF1A1, and treated with camptothecin (0.2 µM) for 18 hours. Equal amounts of whole cell extracts were immunoprecipitated with anti-T7 antibody, resolved by SDS-PAGE and immunoblotted with the indicated antibodies. Parallel sets of inputs (75 µg) were resolved in the same gel to immunoblot with anti-T7 or anti-HA (HA.11) antibodies.

To confirm whether p73 and eEF1A1 interact *in vivo*, cells were transiently co-transfected with plasmids encoding HA-TAp73α and T7-eEF1A1 and whole cell extracts were immunoprecipitated with anti-T7 antibody. HA-TAp73α specifically co-immunoprecipitated with T7-eEF1A1 in both HeLa and SaOS-2 cells (Fig. 1A and data not shown). Furthermore, HA-p53 also co-immunoprecipitated with T7-eEF1A1 (Fig. 1B). These results suggest that both p53 and TAp73α can form a stable complex with eEF1A1 in cells.

In order to determine whether this interaction was specific to TAp73 and p53 we asked whether eEF1A also co-immunoprecipitates with other p73 isoforms or p63. Interestingly, eEF1A1 binding to ΔNp73α, and p63 were detected (Fig. S1 and data not shown). Therefore, eEF1A1 is an interacting partner for all three p53 family paralogues.

### eEF1A1 overexpression inhibits p53-, TAp73α-, and chemotherapy-induced apoptosis

**Figure 2:**
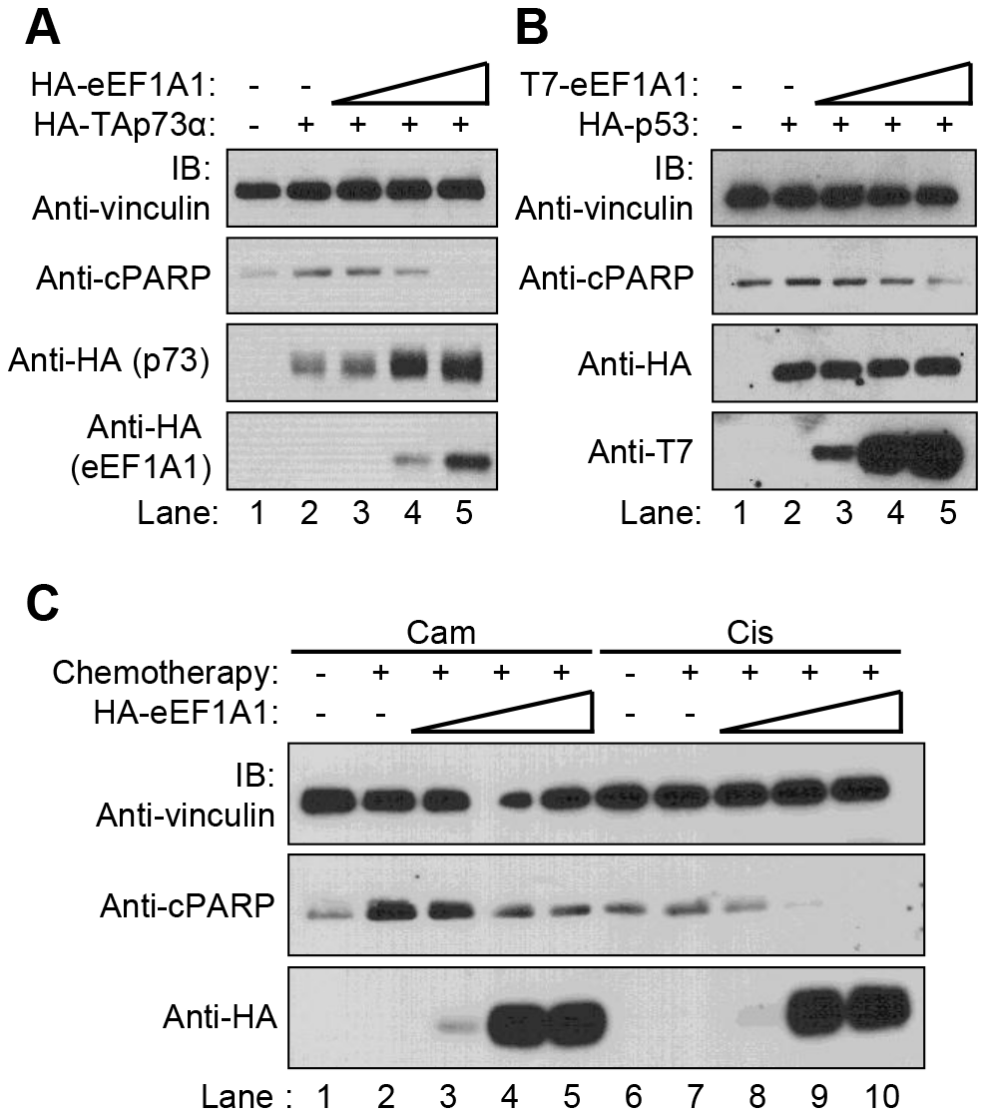
Ectopic expression of eEF1A1 inhibits apoptosis. **Figures 2A–B**, eEF1A1 inhibits p53 and 73-induced apoptosis. HeLa cells were transfected with constant amounts of plasmids encoding HA-p53 or HA-TAp73α and increasing amounts of plasmids encoding T7- or HA-tagged eEF1A1 (p53/73 to eEF1A1 ratios were 1∶1, 1∶5 and 1∶10). Cells were lysed and whole cell extracts were resolved by SDS-PAGE and immunoblotted with the indicated antibodies. In panel A the top anti-HA blot is p73 and the bottom anti-HA blot is eEF1A1. **Figure 2C**, eEF1A1 inhibits chemotherapy-induced apoptosis. HeLa cells were transfected with increasing amounts of plasmids encoding HA-eEF1A1 and treated with camptothecin (5 µM) or cisplatin (2 µM) for 18 hours. Whole cell extracts were resolved by SDS-PAGE and immunoblotted with the indicated antibodies.

Since eEF1A is involved in apoptosis we asked whether eEF1A1 could modulate the pro-apoptotic activity of p53-family proteins. Cells were co-transfected with eEF1A1 together with p53 or TAp73 and the levels of cleaved poly-ADP ribose polymerase (cPARP) were determined as a marker of apoptosis. As expected, in comparison to control mock-transfected cells, overexpression of either p53 or TAp73α alone increased the levels of cPARP (Fig. 2A–B, lane 2 vs lane 1). Co-expression of eEF1A1 resulted in a reduction of p53 and 73-induced cPARP in a dose-dependent manner (Fig. 2A–B, lanes 3 to 5). The inhibition of p53 and 73-induced cPARP was specific to the overexpression of eEF1A1, as over-expression of equivalent amounts of plasmids encoding GFP or eEF1A2 had no effect on the levels of cPARP (Fig. S2). Since the eEF1 isoform eEF1A2 did not affect p53-dependent apoptosis, this suggests that the anti-apoptotic effect of eEF1A1 is independent of its canonical function in translation elongation, which is shared by eEF1A2.

Since p53 and p73 mediate cell death in response to many DNA damaging anti-cancer agents, we asked whether over-expression of eEF1A1 could also inhibit chemotherapy-induced apoptosis. Cells transfected with plasmids encoding eEF1A1 were treated with different chemotherapies (camptothecin or cisplatin). Overexpression of eEF1A1 reduced chemotherapy-induced apoptosis as detected by cPARP in a dose-dependent manner (Fig. 2C). Similar results were observed using etoposide and doxorubicin (data not shown). Taken together, these results demonstrate that overexpression of eEF1A1, but not eEF1A2, inhibits p53-, TAp73α-, and chemotherapy-induced apoptosis.

### eEF1A1 silencing enhances chemotherapy-induced apoptosis

**Figure 3:**
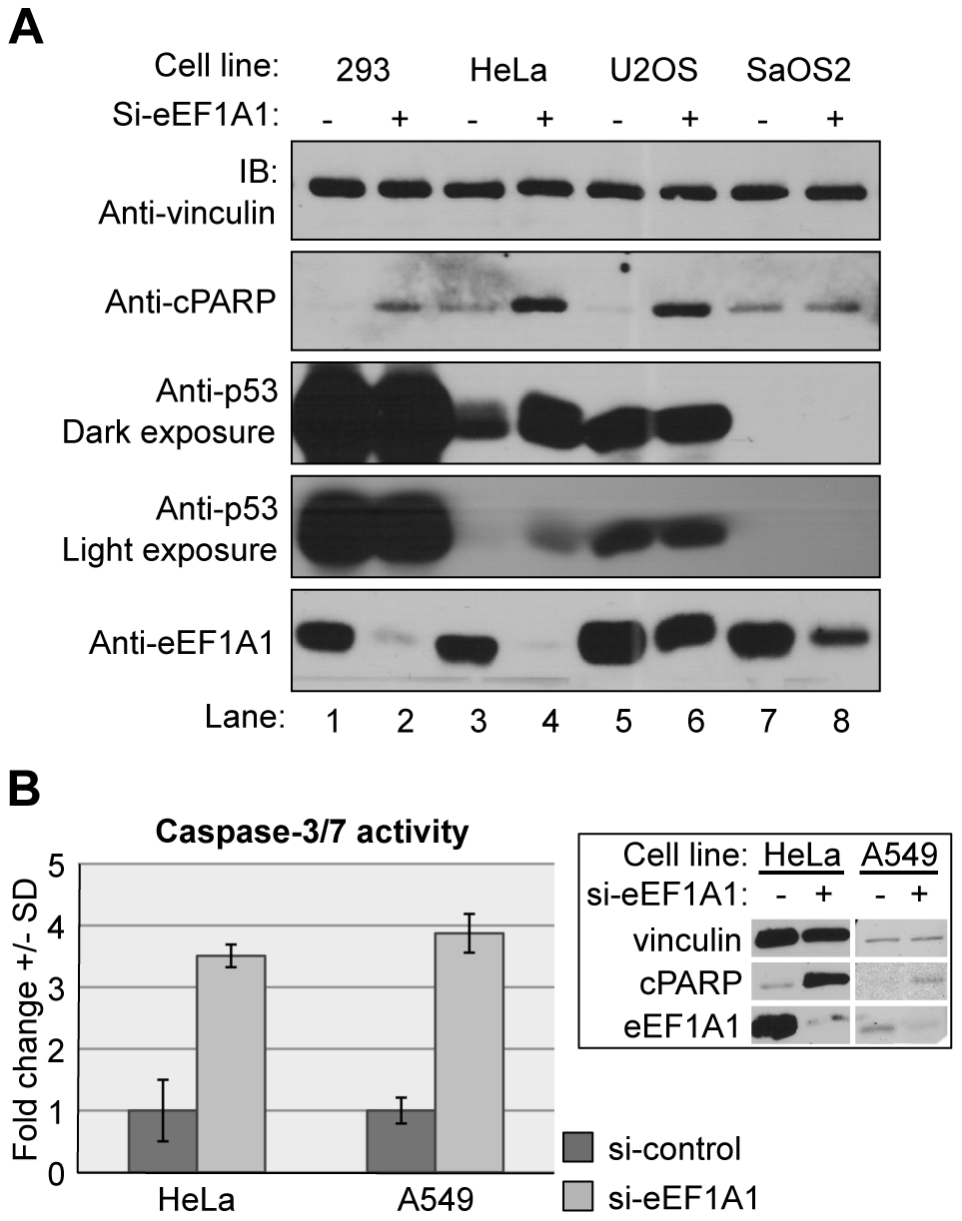
Inhibition of eEF1A1 enhances chemotherapy-induced apoptosis. **Figure 3A**, cells were transfected with siRNA oligonucleotides specific for eEF1A1 or control, and treated with cisplatin (2 µM) for 18 hours. Whole cell extracts were resolved by SDS-PAGE and immunoblotted with the indicated antibodies. **Figure 3B**, HeLa and A549 cells were transfected with siRNA oligonucleotides specific for eEF1A1 or control, treated with cisplatin (2 µM) for 18 hours and subjected to caspase-3/7 activity assays. The activity of control-transfected cells was set to 1 and fold change was plotted. Results are representative of three independent experiments performed in triplicate. Inset: A fraction of the cells were lysed and 25 µg of whole cell extract were immunoblotted with the indicated antibodies.

Since transfection of eEF1A1 inhibited chemotherapy-induced apoptosis, we asked whether siRNA-mediated knockdown of eEF1A1 would sensitize cells to chemotherapy. Cells were transfected with eEF1A1-specific siRNA oligonucleotides and treated with cisplatin. In cell lines with wild-type p53 (HEK293, HeLa, U2OS, A549 and HCT116), inhibition of eEF1A1 increased the levels of cPARP in response to cisplatin treatment (Figs. 3A and S3A). Similar results were observed using other chemotherapies including camptothecin and doxorubicin (Fig. S3C). As a control, results were confirmed using another siRNA that targeted a different eEF1A1 sequence (Fig. S3B). Furthermore, as a second measure of apoptosis, activation of caspases 3 and 7 was measured. eEF1A1 knockdown increased the levels of caspase activity by 3.5 and 3.9 fold in HeLa and A549 cells, respectively (Fig. 3B). Taken together, theses results suggest that inhibition of eEF1A1 sensitizes cells to chemotherapy-induced apoptosis.

### eEF1A1 is a negative regulator of p53 and TAp73

**Figure 4:**
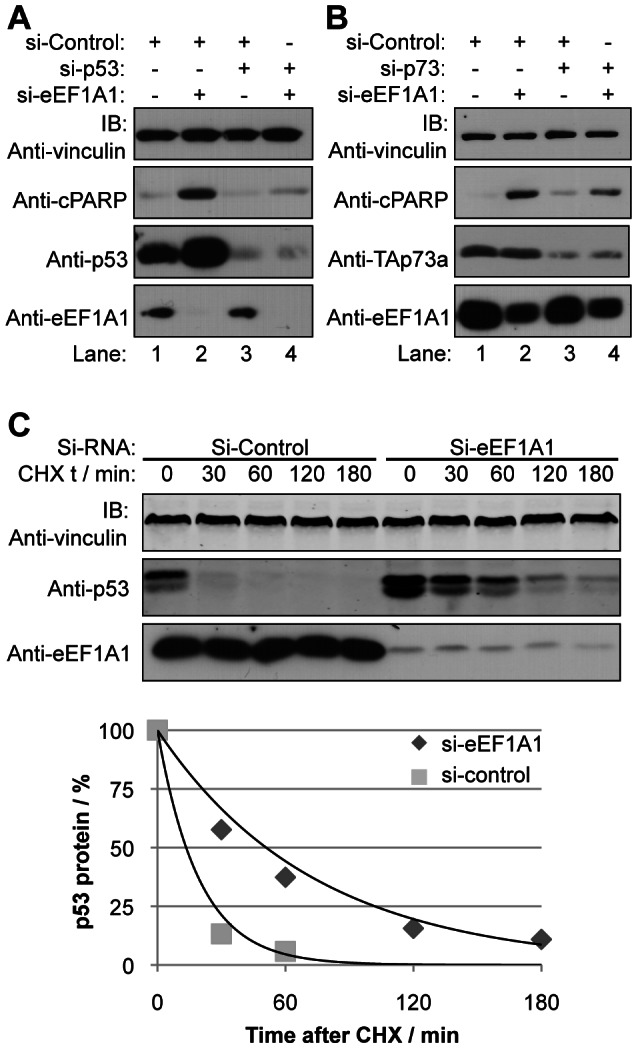
eEF1A1 is a negative regulator of p53 and 73 dependent apoptosis. **Figure 4A–B**, HeLa (panel A) or U2OS (panel B) cells were transfected with siRNA oligonucleotides specific for eEF1A1 and/or p53/73, and treated with cisplatin (2 µM) for 18 hours. Whole cell extracts were resolved by SDS-PAGE and immunoblotted with the indicated antibodies. **Figure 4C**, Inhibition of eEF1A1 increases p53 stability. Hela cells were transfected with siRNA oligonucleotides specific for eEF1A1 or control, and treated with cycloheximide (CHX). Results are representative of three independent experiments. Top panel: Whole cell extracts were collected at the indicated times following CHX treatment, resolved by SDS-PAGE and immunoblotted with the indicated antibodies. Lower panel: The amount of p53 protein relative to vinculin protein was quantified by densitometry and plotted versus the time of cycloheximide treatment. The amount of p53/vinculin at time zero was set to 100%.

Since eEF1A1 binds to p53 and TAp73 and inhibits apoptosis, we next asked whether the anti-apoptotic function of eEF1A1 requires p53 or p73 expression. Inhibition of eEF1A1 increased cisplatin-induced levels of cPARP in different cell lines with wild type *p53*, but eEF1A1 knockdown had no effect on the cPARP levels in *p53-/-* SaOS-2 (Fig. 3A), suggesting that p53 might be necessary to mediate the anti-apoptotic effect of eEF1A1. To determine whether p53 is required we performed siRNA-mediated silencing of both eEF1A1 and p53 in cells treated with cisplatin. As expected, knockdown of eEF1A1 alone led to increased apoptosis as detected by higher levels of cPARP (Fig. 4A, lane 2), while p53 siRNA resulted in resistance to cisplatin with decreased levels of cleaved PARP (lane 3). Interestingly, siRNA knockdown of both eEF1A1 and p53 partially rescued the apoptosis observed with eEF1A1 single knockdown in Hela cells (Fig. 4A, lane 4). Similar results were obtained in HEK293 cells (Fig. S4A). To determine whether like p53, TAp73 is required for eEF1A1 anti-apoptotic function, similar experiments were performed in which both eEF1A1 and TAp73 were silenced simultaneously (Figs. 4B and S4B). Silencing of both TAp73 and eEF1A1 also partially rescued the death observed in cells in which only eEF1A1 is silenced. These results suggest that eEF1A1 is a negative regulator of the pro-apoptotic function of p53 and TAp73.

Many proteins that interact with and regulate the apoptotic function of p53 and p73 affect the stability of these proteins. Interestingly, we noticed that inhibition of eEF1A1 markedly increased the steady state levels of p53 in HeLa cells (Figs. 3A and S3A). In contrast to effects on p53 protein, eEF1A1 knockdown did not affect the levels of *p53* mRNA (Fig. S4C). Furthermore, eEF1A1 siRNA increased the half-life of p53 from 14 to 51 minutes in HeLa cells (Fig. 4C). However, cycloheximide experiments failed to show significant changes in protein stability in other cell lines tested, including SiHa and CaSki cervical cancer cell lines that, like HeLa, contain human papilloma virus (data not shown). We also did not detect changes in p73 steady state levels following eEF1A1 knockdown (Fig. 4B). These data suggest that destabilization of p53 may be a mechanism by which eEF1A1 affects p53-dependent apoptosis in HeLa cells, however, this does not explain its anti-apoptotic role in other cellular contexts. Furthermore, eEF1A1 inhibition of p73-dependent apoptosis is not associated with decreased p73 protein levels. Thus, the negative regulation of p53 family proteins by eEF1A1 is not due to changes in protein stability.

## Discussion

The p53 family proteins form a network that controls cell proliferation and death in response to stresses such as DNA damage. Although many p53 interacting proteins have been described, only a subset binds to and regulates p63 and p73 [1], [10]. Since p73 can induce apoptosis independent of p53, and p73 is rarely mutated in cancers, elucidation of p73-dependent cell death pathways in response to chemotherapies may lead to the identification of novel drug targets for tumors with or without p53 aberrations [40]. In this study, we have identified eEF1A1 as a novel interacting partner of p73. We have demonstrated that eEF1A1 does not selectively bind p73, but instead can form complexes in vivo with all three p53 family proteins. Overexpression and silencing of eEF1A1 results in chemoresistance and enhanced chemosensitivity, respectively. Experiments in cells lacking *p53* and simultaneous silencing of eEF1A1 and p53 or p73 suggest that eEF1A1 inhibition of apoptosis is, at least in part, mediated through p53 or p73. siRNA knockdown of eEF1A1 increased chemosensitivity in cell lines with wild type *p53*, but had no effect in *p53^−/−^* cells. Furthermore, double knockdown of p53/73 and eEF1A1 partially rescued the chemoresistance observed in cells with p53 or 73 knockdown. Our findings suggest that the anti-apoptotic effects of eEF1A1 are, in part, related to its effect on p53 family proteins.

There are many potential molecular mechanisms by which eEF1A1 may inhibit p53 and p73. Many p53 interacting proteins modulate p53 stability by promoting or inhibiting post-translational modifications that lead to degradation. We found that eEF1A1 reduced p53 steady state levels and promoted p53 degradation in HeLa cells, but did not affect the half-life of p53 in the majority of cells tested, including other cervical cancer cell lines that, like Hela, contain human papilloma virus. Taken together, these data suggest that eEF1A1 effects are not due to modulating p53 degradation in most cancer cells. Interestingly, eEF1A1 transfection resulted in decreased steady state p53 levels but increased p73 levels (Fig. 2). Similar results have been described for HDM2. HDM2 binds to and inhibits the transcriptional activity of both p53 and p73; however, it only promotes ubiquitination and degradation of p53, and instead results in increased steady state levels of p73 [43]. Furthermore, HDM2 has also been shown to bind to eEF1A [44]. Thus, it is possible that eEF1A effects on p73 and p53 may also be indirectly mediated by its binding to HDM2.

Other mechanisms by which p53-family interactors such as HDM2 modulate activity, which are independent of effects on stability, include effects on nuclear-cytoplasmic shuttling and transcriptional activity. eEF1A has been shown to bind to and promote nuclear export of the von Hippel-Lindau (VHL) tumor suppressor and the polyA-binding protein 1 (PABP1) [41]. Interestingly, *EEF1A* was shown to be up-regulated by p53 [14], suggesting a potential autoregulatory feedback loop between p53 and eEF1A1. Similar feedback loops are commonly involved in the regulation of p53 and p73 by HDM2, Pirh2, Itch, cyclin G and DEC1, by both ubiquitin-dependent and –independent mechanisms [45], [46], [47], [48], [49]. Although we did not detect consistent effects on p53 subcellular localization by immunofluorescence or fractionation following eEF1A1 inhibition (data not shown), we did observe increased levels of the p53 and p73 target p21 (Fig. S3A), suggesting that eEF1A1 anti-apoptotic function may be in part due to inhibition of p53-mediated transactivation of target genes.

Previous studies have concluded that eEF1A1 has anti-apoptotic functions in cancer cell models but the mechanisms were unclear [29], [31], [32], [34], [35]. Our findings demonstrate that the pro-survival activity of eEF1A1 following chemotherapy treatment may be in part due to regulation of p73 and p53. Interestingly, the regulation of p53 and p73 seems to be independent of the canonical function of eEF1A1 in protein translation, as eEF1A2, which is fully interchangeable in its canonical translation elongation function with eEF1A1, did not affect apoptosis (Fig. S2B). Following treatment with the protein synthesis inhibitor cycloheximide, eEF1A1 still reduced p53 levels and activities, further supporting a translation-independent function. Notably, eEF1A is not the only translation factor involved in tumorigenesis. Increased expression of the eukaryotic translation initiation factor eIF4E is also associated with tumors in the breast, colon, larynx and lungs [50], [51], [52]. Furthermore, the prostate tumor-inducing gene 1 (PTI-1), which has oncogenic properties and is expressed in cancer, but not normal cell lines, shares significant homology with eEF1A1 [53].

Recently, the development of therapies to restore p53 function has focused on blocking inhibitory interactions. The prototype for these drugs has been nutlin, which disrupts the interaction between p53 and HDM2, reducing p53 degradation and enhancing p53 function, and interestingly, can also inhibit p73-HDM2 complexes [54], [55], [56], [57], [58]. Thus, while inhibiting the expression of eEF1A1, which is critical for translation, is unlikely to be feasible, targeting the interaction between eEF1A1 and the pro-apoptotic p53 family proteins such as p53 and TAp73 may lead to apoptosis of tumor cells as well as enhance sensitivity to common chemotherapies. Further studies to elucidate the functional importance of the eEF1A1-p53 and -TAp73 interactions, and the identification of other important p53 family regulatory proteins, may provide additional insight into potential novel targets for small molecule inhibitors that may sensitize tumors to chemotherapy.

## Supporting Information

Figure S1 **eEF1A1 interacts with p63.** HeLa cells were transfected with plasmids encoding Flag-TAp63α and HA-eEF1A1, and treated with camptothecin (0.2 µM) for 18 hours. Equal amounts of whole cell extracts were immunoprecipitated with anti-HA (12CA5) antibody, resolved by SDS-PAGE and immunoblotted with anti-Flag and anti-HA (HA.11) antibodies.
(PDF)

Figure S2 **Ectopic expression of eEF1A1 inhibits p53 and p73 induced apoptosis.** **Figure S2A**, HeLa cells were transfected with constant amounts of plasmid encoding HA-TAp73α and increasing amounts of plasmid encoding either HA-eEF1A1 or GFP (p73 to eEF1A1/GFP ratios were 1∶1, 1∶5 and 1∶10). Cells were lysed and whole cell extracts were resolved by SDS-PAGE and immunoblotted with the indicated antibodies. **Figure S2B**, HeLa cells were transfected with constant amounts of plasmid encoding HA-p53 and increasing amounts of plasmid encoding either HA tagged eEF1A1 or eEF1A2 (p53 to eEF1A1/2 ratios were 1∶1, 1∶5 and 1∶10). Cells were lysed and whole cell extracts were resolved by SDS-PAGE and immunoblotted with the indicated antibodies.
(PDF)

Figure S3 **Inhibition of eEF1A1 enhances chemotherapy-induced apoptosis.** **Figure S3A**, cells were transfected with siRNA oligonucleotides specific for eEF1A1 or control, and treated with cisplatin (2 µM) for 18 hours. Whole cell extracts were resolved by SDS-PAGE and immunoblotted with the indicated antibodies. **Figure S3B**, HeLa cells were transfected with two different siRNA oligonucleotides specific for eEF1A1 or control. Cells were treated, or not, with cisplatinum (2 µM) for 18 hours. Whole cell extracts were resolved by SDS-PAGE and immunoblotted with the indicated antibodies. **Figure S3C**, HeLa cells were transfected with siRNA oligonucleotides specific for eEF1A1 or control, and treated with doxorubicin (1 µM) or camptothecin (3 µM) for 18 hours. Whole cell extracts were resolved by SDS-PAGE and immunoblotted with the indicated antibodies.
(PDF)

Figure S4 **eEF1A1 is a negative regulator of p53 and p73 dependent apoptosis.** HEK293 cells were transfected with siRNA oligonucleotides specific for eEF1A1 and/or p53 (panel A) or p73 (panel B), and treated with cisplatin (2 µM) for 18 hours. Whole cell extracts were resolved by SDS-PAGE and immunoblotted with the indicated antibodies. **Figure S4C**, HeLa cells were transfected with two different siRNA oligonucleotides specific for eEF1A1 or control. RNA was isolated and subjected to RT-PCR using the indicated primers. A fraction of cells were lysed and whole cell extracts were immunoblotted with the indicated antibodies.
(PDF)

## References

[1] Murray-Zmijewski F, Lane DP, Bourdon JC (2006) p53/p63/p73 isoforms: an orchestra of isoforms to harmonise cell differentiation and response to stress. Cell Death Differ 13: 962–972. 16601753 10.1038/sj.cdd.4401914

[2] Marin MC, Jost CA, Irwin MS, DeCaprio JA, Caput D, et al (1998) Viral oncoproteins discriminate between p53 and the p53 homolog p73. Mol Cell Biol 18: 6316–6324. 9774648 10.1128/mcb.18.11.6316 PMC109218

[3] Pozniak CD, Radinovic S, Yang A, McKeon F, Kaplan DR, et al (2000) An anti-apoptotic role for the p53 family member, p73, during developmental neuron death. Science 289: 304–306. 10894779 10.1126/science.289.5477.304

[4] Zaika AI, Slade N, Erster SH, Sansome C, Joseph TW, et al (2002) DeltaNp73, a dominant-negative inhibitor of wild-type p53 and TAp73, is up-regulated in human tumors. J Exp Med 196: 765–780. 12235210 10.1084/jem.20020179 PMC2194062

[5] Kruse JP, Gu W (2009) Modes of p53 regulation. Cell 137: 609–622. 19450511 10.1016/j.cell.2009.04.050 PMC3737742

[6] Irwin MS (2004) Family feud in chemosensitvity: p73 and mutant p53. Cell Cycle 3: 319–323. 14739781

[7] Rocco JW, Leong CO, Kuperwasser N, DeYoung MP, Ellisen LW (2006) p63 mediates survival in squamous cell carcinoma by suppression of p73-dependent apoptosis. Cancer Cell 9: 45–56. 16413471 10.1016/j.ccr.2005.12.013

[8] Wilhelm MT, Rufini A, Wetzel MK, Tsuchihara K, Inoue S, et al (2010) Isoform-specific p73 knockout mice reveal a novel role for delta Np73 in the DNA damage response pathway. Genes Dev 24: 549–560. 20194434 10.1101/gad.1873910 PMC2841333

[9] Aylon Y, Oren M (2007) Living with p53, dying of p53. Cell 130: 597–600. 17719538 10.1016/j.cell.2007.08.005

[10] Collavin L, Lunardi A, Del Sal G (2010) p53-family proteins and their regulators: hubs and spokes in tumor suppression. Cell Death Differ 17: 901–911. 20379196 10.1038/cdd.2010.35

[11] Hershey JW (1991) Translational control in mammalian cells. Annu Rev Biochem 60: 717–755. 1883206 10.1146/annurev.bi.60.070191.003441

[12] Thornton S, Anand N, Purcell D, Lee J (2003) Not just for housekeeping: protein initiation and elongation factors in cell growth and tumorigenesis. J Mol Med 81: 536–548. 12898041 10.1007/s00109-003-0461-8

[13] Tatsuka M, Mitsui H, Wada M, Nagata A, Nojima H, et al (1992) Elongation factor-1 alpha gene determines susceptibility to transformation. Nature 359: 333–336. 1383827 10.1038/359333a0

[14] Kato MV, Sato H, Nagayoshi M, Ikawa Y (1997) Upregulation of the elongation factor-1alpha gene by p53 in association with death of an erythroleukemic cell line. Blood 90: 1373–1378. 9269753

[15] Lamberti A, Caraglia M, Longo O, Marra M, Abbruzzese A, et al (2004) The translation elongation factor 1A in tumorigenesis, signal transduction and apoptosis: review article. Amino Acids 26: 443–448. 15290352 10.1007/s00726-004-0088-2

[16] Al-Maghrebi M, Anim JT, Olalu AA (2005) Up-regulation of eukaryotic elongation factor-1 subunits in breast carcinoma. Anticancer Res 25: 2573–2577. 16080495

[17] Chuang SM, Chen L, Lambertson D, Anand M, Kinzy TG, et al (2005) Proteasome-mediated degradation of cotranslationally damaged proteins involves translation elongation factor 1A. Mol Cell Biol 25: 403–413. 15601860 10.1128/MCB.25.1.403-413.2005 PMC538794

[18] Gross SR, Kinzy TG (2005) Translation elongation factor 1A is essential for regulation of the actin cytoskeleton and cell morphology. Nat Struct Mol Biol 12: 772–778. 16116436 10.1038/nsmb979

[19] Grant AG, Flomen RM, Tizard ML, Grant DA (1992) Differential screening of a human pancreatic adenocarcinoma lambda gt11 expression library has identified increased transcription of elongation factor EF-1 alpha in tumour cells. Int J Cancer 50: 740–745. 1544708 10.1002/ijc.2910500513

[20] Zhang L, Zhou W, Velculescu VE, Kern SE, Hruban RH, et al (1997) Gene expression profiles in normal and cancer cells. Science 276: 1268–1272. 9157888 10.1126/science.276.5316.1268

[21] Xie D, Jauch A, Miller CW, Bartram CR, Koeffler HP (2002) Discovery of over-expressed genes and genetic alterations in breast cancer cells using a combination of suppression subtractive hybridization, multiplex FISH and comparative genomic hybridization. Int J Oncol 21: 499–507. 12168092

[22] Mohler JL, Morris TL, Ford OH 3rd, Alvey RF, Sakamoto C, et al (2002) Identification of differentially expressed genes associated with androgen-independent growth of prostate cancer. Prostate 51: 247–255. 11987153 10.1002/pros.10086

[23] de Wit NJ, Burtscher HJ, Weidle UH, Ruiter DJ, van Muijen GN (2002) Differentially expressed genes identified in human melanoma cell lines with different metastatic behaviour using high density oligonucleotide arrays. Melanoma Res 12: 57–69. 11828259 10.1097/00008390-200202000-00009

[24] Johnsson A, Zeelenberg I, Min Y, Hilinski J, Berry C, et al (2000) Identification of genes differentially expressed in association with acquired cisplatin resistance. Br J Cancer 83: 1047–1054. 10993653 10.1054/bjoc.2000.1420 PMC2363570

[25] Edmonds BT, Wyckoff J, Yeung YG, Wang Y, Stanley ER, et al (1996) Elongation factor-1 alpha is an overexpressed actin binding protein in metastatic rat mammary adenocarcinoma. J Cell Sci 109 (Pt 11) 2705–2714. 8937988 10.1242/jcs.109.11.2705

[26] Duttaroy A, Bourbeau D, Wang XL, Wang E (1998) Apoptosis rate can be accelerated or decelerated by overexpression or reduction of the level of elongation factor-1 alpha. Exp Cell Res 238: 168–176. 9457069 10.1006/excr.1997.3819

[27] Chen E, Proestou G, Bourbeau D, Wang E (2000) Rapid up-regulation of peptide elongation factor EF-1alpha protein levels is an immediate early event during oxidative stress-induced apoptosis. Exp Cell Res 259: 140–148. 10942586 10.1006/excr.2000.4952

[28] Borradaile NM, Buhman KK, Listenberger LL, Magee CJ, Morimoto ET, et al (2006) A critical role for eukaryotic elongation factor 1A-1 in lipotoxic cell death. Mol Biol Cell 17: 770–778. 16319173 10.1091/mbc.E05-08-0742 PMC1356587

[29] Lamberti A, Longo O, Marra M, Tagliaferri P, Bismuto E, et al (2007) C-Raf antagonizes apoptosis induced by IFN-alpha in human lung cancer cells by phosphorylation and increase of the intracellular content of elongation factor 1A. Cell Death Differ 14: 952–962. 17332776 10.1038/sj.cdd.4402102

[30] Kobayashi Y, Yonehara S (2009) Novel cell death by downregulation of eEF1A1 expression in tetraploids. Cell Death Differ 16: 139–150. 18820646 10.1038/cdd.2008.136

[31] Talapatra S, Wagner JD, Thompson CB (2002) Elongation factor-1 alpha is a selective regulator of growth factor withdrawal and ER stress-induced apoptosis. Cell Death Differ 9: 856–861. 12107828 10.1038/sj.cdd.4401078

[32] Pecorari L, Marin O, Silvestri C, Candini O, Rossi E, et al (2009) Elongation Factor 1 alpha interacts with phospho-Akt in breast cancer cells and regulates their proliferation, survival and motility. Mol Cancer 8: 58. 19646290 10.1186/1476-4598-8-58 PMC2727493

[33] Kim J, Namkung W, Yoon JS, Jo MJ, Lee SH, et al (2009) The role of translation elongation factor eEF1A in intracellular alkalinization-induced tumor cell growth. Lab Invest 89: 867–874. 19506553 10.1038/labinvest.2009.53

[34] Selga E, Oleaga C, Ramirez S, de Almagro MC, Noe V, et al (2009) Networking of differentially expressed genes in human cancer cells resistant to methotrexate. Genome Med 1: 83. 10.1186/gm83 PMC2768990 19732436

[35] Ruest LB, Marcotte R, Wang E (2002) Peptide elongation factor eEF1A-2/S1 expression in cultured differentiated myotubes and its protective effect against caspase-3-mediated apoptosis. J Biol Chem 277: 5418–5425. 11724805 10.1074/jbc.M110685200 PMC2803684

[36] Bunz F, Dutriaux A, Lengauer C, Waldman T, Zhou S, et al (1998) Requirement for p53 and p21 to sustain G2 arrest after DNA damage. Science 282: 1497–1501. 9822382 10.1126/science.282.5393.1497

[37] Irwin M, Marin MC, Phillips AC, Seelan RS, Smith DI, et al (2000) Role for the p53 homologue p73 in E2F-1-induced apoptosis. Nature 407: 645–648. 11034215 10.1038/35036614

[38] Chung J, Lau J, Cheng LS, Grant RI, Robinson F, et al (2010) SATB2 augments DeltaNp63alpha in head and neck squamous cell carcinoma. EMBO Rep 11: 777–783. 20829881 10.1038/embor.2010.125 PMC2948183

[39] Lau L, Hansford LM, Cheng LS, Hang M, Baruchel S, et al (2007) Cyclooxygenase inhibitors modulate the p53/HDM2 pathway and enhance chemotherapy-induced apoptosis in neuroblastoma. Oncogene 26: 1920–1931. 16983334 10.1038/sj.onc.1209981

[40] Irwin MS, Kondo K, Marin MC, Cheng LS, Hahn WC, et al (2003) Chemosensitivity linked to p73 function. Cancer Cell 3: 403–410. 12726865 10.1016/s1535-6108(03)00078-3

[41] Khacho M, Mekhail K, Pilon-Larose K, Pause A, Cote J, et al (2008) eEF1A is a novel component of the mammalian nuclear protein export machinery. Mol Biol Cell 19: 5296–5308. 18799616 10.1091/mbc.E08-06-0562 PMC2592675

[42] Watson IR, Blanch A, Lin DC, Ohh M, Irwin MS (2006) Mdm2-mediated NEDD8 modification of TAp73 regulates its transactivation function. J Biol Chem 281: 34096–34103. 16980297 10.1074/jbc.M603654200

[43] Balint E, Bates S, Vousden KH (1999) Mdm2 binds p73 alpha without targeting degradation. Oncogene 18: 3923–3929. 10435614 10.1038/sj.onc.1202781

[44] Frum R, Busby SA, Ramamoorthy M, Deb S, Shabanowitz J, et al (2007) HDM2-binding partners: interaction with translation elongation factor EF1alpha. J Proteome Res 6: 1410–1417. 17373842 10.1021/pr060584p PMC4626875

[45] Momand J, Zambetti GP, Olson DC, George D, Levine AJ (1992) The mdm-2 oncogene product forms a complex with the p53 protein and inhibits p53-mediated transactivation. Cell 69: 1237–1245. 1535557 10.1016/0092-8674(92)90644-r

[46] Leng RP, Lin Y, Ma W, Wu H, Lemmers B, et al (2003) Pirh2, a p53-induced ubiquitin-protein ligase, promotes p53 degradation. Cell 112: 779–791. 12654245 10.1016/s0092-8674(03)00193-4

[47] Rossi M, De Laurenzi V, Munarriz E, Green DR, Liu YC, et al (2005) The ubiquitin-protein ligase Itch regulates p73 stability. EMBO J 24: 836–848. 15678106 10.1038/sj.emboj.7600444 PMC549609

[48] Ohtsuka T, Ryu H, Minamishima YA, Ryo A, Lee SW (2003) Modulation of p53 and p73 levels by cyclin G: implication of a negative feedback regulation. Oncogene 22: 1678–1687. 12642871 10.1038/sj.onc.1206306

[49] Qian Y, Jung YS, Chen X (2012) DEC1 and MIC-1: new players of p53-dependent cell fate decision. Cell Cycle 11: 3525–3526. 22935706 10.4161/cc.21962 PMC3478296

[50] Li BD, Gruner JS, Abreo F, Johnson LW, Yu H, et al (2002) Prospective study of eukaryotic initiation factor 4E protein elevation and breast cancer outcome. Ann Surg 235: 732–738 discussion 738–739. 11981220 10.1097/00000658-200205000-00016 PMC1422500

[51] Nathan CO, Sanders K, Abreo FW, Nassar R, Glass J (2000) Correlation of p53 and the proto-oncogene eIF4E in larynx cancers: prognostic implications. Cancer Res 60: 3599–3604. 10910074

[52] Rosenwald IB, Hutzler MJ, Wang S, Savas L, Fraire AE (2001) Expression of eukaryotic translation initiation factors 4E and 2alpha is increased frequently in bronchioloalveolar but not in squamous cell carcinomas of the lung. Cancer 92: 2164–2171. 11596034 10.1002/1097-0142(20011015)92:8<2164::aid-cncr1559>3.0.co;2-a

[53] Gopalkrishnan RV, Su ZZ, Goldstein NI, Fisher PB (1999) Translational infidelity and human cancer: role of the PTI-1 oncogene. Int J Biochem Cell Biol 31: 151–162. 10216950 10.1016/s1357-2725(98)00138-1

[54] Vassilev LT, Vu BT, Graves B, Carvajal D, Podlaski F, et al (2004) In vivo activation of the p53 pathway by small-molecule antagonists of MDM2. Science 303: 844–848. 14704432 10.1126/science.1092472

[55] Tovar C, Rosinski J, Filipovic Z, Higgins B, Kolinsky K, et al (2006) Small-molecule MDM2 antagonists reveal aberrant p53 signaling in cancer: implications for therapy. Proc Natl Acad Sci U S A 103: 1888–1893. 16443686 10.1073/pnas.0507493103 PMC1413632

[56] Van Maerken T, Speleman F, Vermeulen J, Lambertz I, De Clercq S, et al (2006) Small-molecule MDM2 antagonists as a new therapy concept for neuroblastoma. Cancer Res 66: 9646–9655. 17018622 10.1158/0008-5472.CAN-06-0792

[57] Laurie NA, Donovan SL, Shih CS, Zhang J, Mills N, et al (2006) Inactivation of the p53 pathway in retinoblastoma. Nature 444: 61–66. 17080083 10.1038/nature05194

[58] Coll-Mulet L, Iglesias-Serret D, Santidrian AF, Cosialls AM, de Frias M, et al (2006) MDM2 antagonists activate p53 and synergize with genotoxic drugs in B-cell chronic lymphocytic leukemia cells. Blood 107: 4109–4114. 16439685 10.1182/blood-2005-08-3273

