# Supplementary material for: Eukaryotic Translation Elongation Factor 1-Alpha 1 Inhibits p53 and p73 Dependent Apoptosis and Chemotherapy Sensitivity
Source: PLoS One. 2013 Jun 14;8(6):e66436. doi: 10.1371/journal.pone.0066436 (PMC3682968; doi:10.1371/journal.pone.0066436)
Supplement: Figure S1 — eEF1A1 interacts with p63. HeLa cells were transfected with plasmids encoding Flag-TAp63α and HA-eEF1A1, and treated with camptothecin (0.2 µM) for 18 hours. Equal amounts of whole cell extracts were immunoprecipitated with anti-HA (12CA5) antibody, resolved by SDS-PAGE and immunoblotted with anti-Flag and anti-HA (HA.11) antibodies. (PDF) [file pone.0066436.s001.pdf]

Supplemental Figure S1. Blanch *et al*.

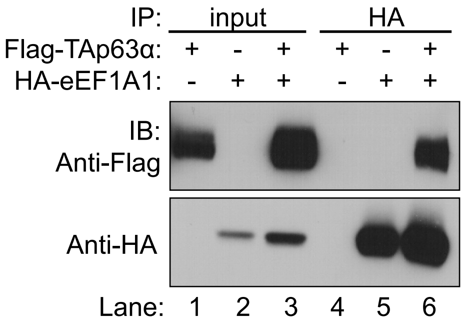

Figure S1. eEF1A1 interacts with p63. HeLa cells were transfected with plasmids encoding Flag-TAp63α and HA-eEF1A1, and treated with camptothecin (0.2 μM) for 18 hours. Equal amounts of whole cell extracts were immunoprecipitated with anti-HA (12CA5) antibody, resolved by SDS-PAGE and immunoblotted with anti-Flag and anti-HA (HA.11) antibodies.
