# Supplementary material for: Eukaryotic Translation Elongation Factor 1-Alpha 1 Inhibits p53 and p73 Dependent Apoptosis and Chemotherapy Sensitivity
Source: PLoS One. 2013 Jun 14;8(6):e66436. doi: 10.1371/journal.pone.0066436 (PMC3682968; doi:10.1371/journal.pone.0066436)
Supplement: Figure S2 — Ectopic expression of eEF1A1 inhibits p53 and p73 induced apoptosis. Figure S2A, HeLa cells were transfected with constant amounts of plasmid encoding HA-TAp73α and increasing amounts of plasmid encoding either HA-eEF1A1 or GFP (p73 to eEF1A1/GFP ratios were 1∶1, 1∶5 and 1∶10). Cells were lysed and whole cell extracts were resolved by SDS-PAGE and immunoblotted with the indicated antibodies. Figure S2B, HeLa cells were transfected with constant amounts of plasmid encoding HA-p53 and increasing amounts of plasmid encoding either HA tagged eEF1A1 or eEF1A2 (p53 to eEF1A1/2 ratios were 1∶1, 1∶5 and 1∶10). Cells were lysed and whole cell extracts were resolved by SDS-PAGE and immunoblotted with the indicated antibodies. (PDF) [file pone.0066436.s002.pdf]

Supplemental Figure S2. Blanch *et al*.

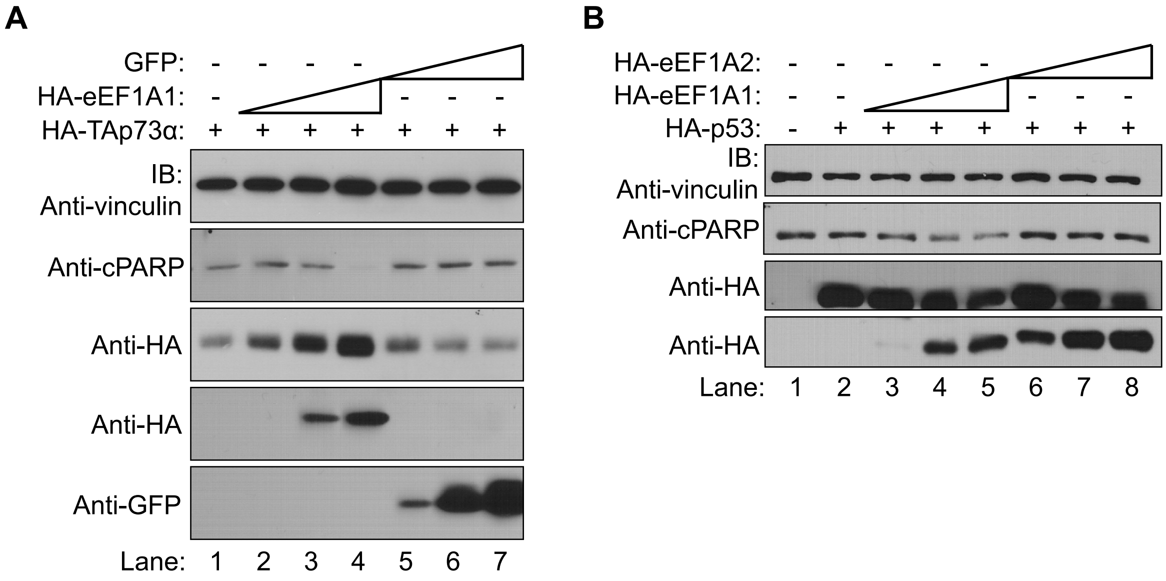

Figure S2. Ectopic expression of eEF1A1 inhibits p53 and p73 induced apoptosis. Figure S2A, HeLa cells were transfected with constant amounts of plasmid encoding HA-TAp73α and increasing amounts of plasmid encoding either HA-eEF1A1 or GFP (p73 to eEF1A1/GFP ratios were 1:1, 1:5 and 1:10). Cells were lysed and whole cell extracts were resolved by SDS-PAGE and immunoblotted with the indicated antibodies. **Figure S2B,** HeLa cells were transfected with constant amounts of plasmid encoding HA-p53 and increasing amounts of plasmid encoding either HA tagged eEF1A1 or eEF1A2 (p53 to eEF1A1/2 ratios were 1:1, 1:5 and 1:10). Cells were lysed and whole cell extracts were resolved by SDS-PAGE and immunoblotted with the indicated antibodies.
