# Supplementary material for: Eukaryotic Translation Elongation Factor 1-Alpha 1 Inhibits p53 and p73 Dependent Apoptosis and Chemotherapy Sensitivity
Source: PLoS One. 2013 Jun 14;8(6):e66436. doi: 10.1371/journal.pone.0066436 (PMC3682968; doi:10.1371/journal.pone.0066436)
Supplement: Figure S3 — Inhibition of eEF1A1 enhances chemotherapy-induced apoptosis. Figure S3A, cells were transfected with siRNA oligonucleotides specific for eEF1A1 or control, and treated with cisplatin (2 µM) for 18 hours. Whole cell extracts were resolved by SDS-PAGE and immunoblotted with the indicated antibodies. Figure S3B, HeLa cells were transfected with two different siRNA oligonucleotides specific for eEF1A1 or control. Cells were treated, or not, with cisplatinum (2 µM) for 18 hours. Whole cell extracts were resolved by SDS-PAGE and immunoblotted with the indicated antibodies. Figure S3C, HeLa cells were transfected with siRNA oligonucleotides specific for eEF1A1 or control, and treated with doxorubicin (1 µM) or camptothecin (3 µM) for 18 hours. Whole cell extracts were resolved by SDS-PAGE and immunoblotted with the indicated antibodies. (PDF) [file pone.0066436.s003.pdf]

Supplemental Figure S3. Blanch *et al*.

A

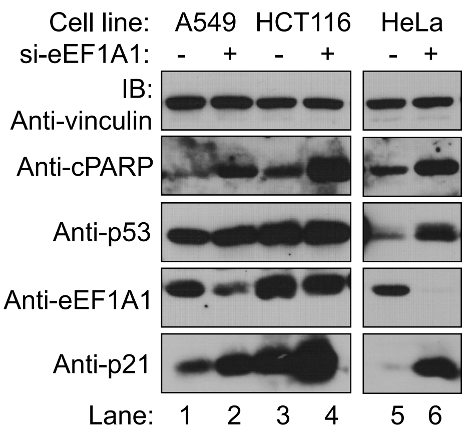

B

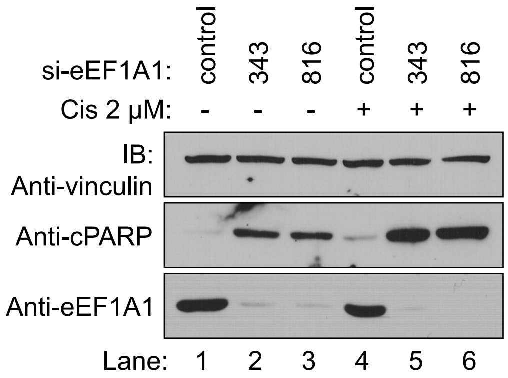

C

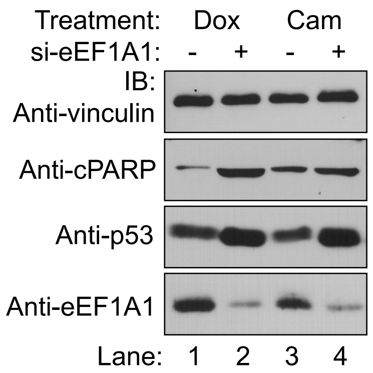

Figure S3. Inhibition of eEF1A1 enhances chemotherapy-induced apoptosis. Figure S3A, cells were transfected with siRNA oligonucleotides specific for eEF1A1 or control, and treated with cisplatin (2 µM) for 18 hours. Whole cell extracts were resolved by SDS-PAGE and immunoblotted with the indicated antibodies. **Figure S3B,** HeLa cells were transfected with two different siRNA oligonucleotides specific for eEF1A1 or control. Cells were treated, or not, with cisplatinum (2 µM) for 18 hours. Whole cell extracts were resolved by SDS-PAGE and immunoblotted with the indicated antibodies. **Figure S3C,** HeLa cells were transfected with siRNA oligonucleotides specific for eEF1A1 or control, and treated with doxorubicin (1 µM) or camptothecin (3 µM) for 18 hours. Whole cell extracts were resolved by SDS-PAGE and immunoblotted with the indicated antibodies.
