# Supplementary material for: Eukaryotic Translation Elongation Factor 1-Alpha 1 Inhibits p53 and p73 Dependent Apoptosis and Chemotherapy Sensitivity
Source: PLoS One. 2013 Jun 14;8(6):e66436. doi: 10.1371/journal.pone.0066436 (PMC3682968; doi:10.1371/journal.pone.0066436)
Supplement: Figure S4 — eEF1A1 is a negative regulator of p53 and p73 dependent apoptosis. HEK293 cells were transfected with siRNA oligonucleotides specific for eEF1A1 and/or p53 (panel A) or p73 (panel B), and treated with cisplatin (2 µM) for 18 hours. Whole cell extracts were resolved by SDS-PAGE and immunoblotted with the indicated antibodies. Figure S4C, HeLa cells were transfected with two different siRNA oligonucleotides specific for eEF1A1 or control. RNA was isolated and subjected to RT-PCR using the indicated primers. A fraction of cells were lysed and whole cell extracts were immunoblotted with the indicated antibodies. (PDF) [file pone.0066436.s004.pdf]

Supplemental Figure S4. Blanch *et al*.

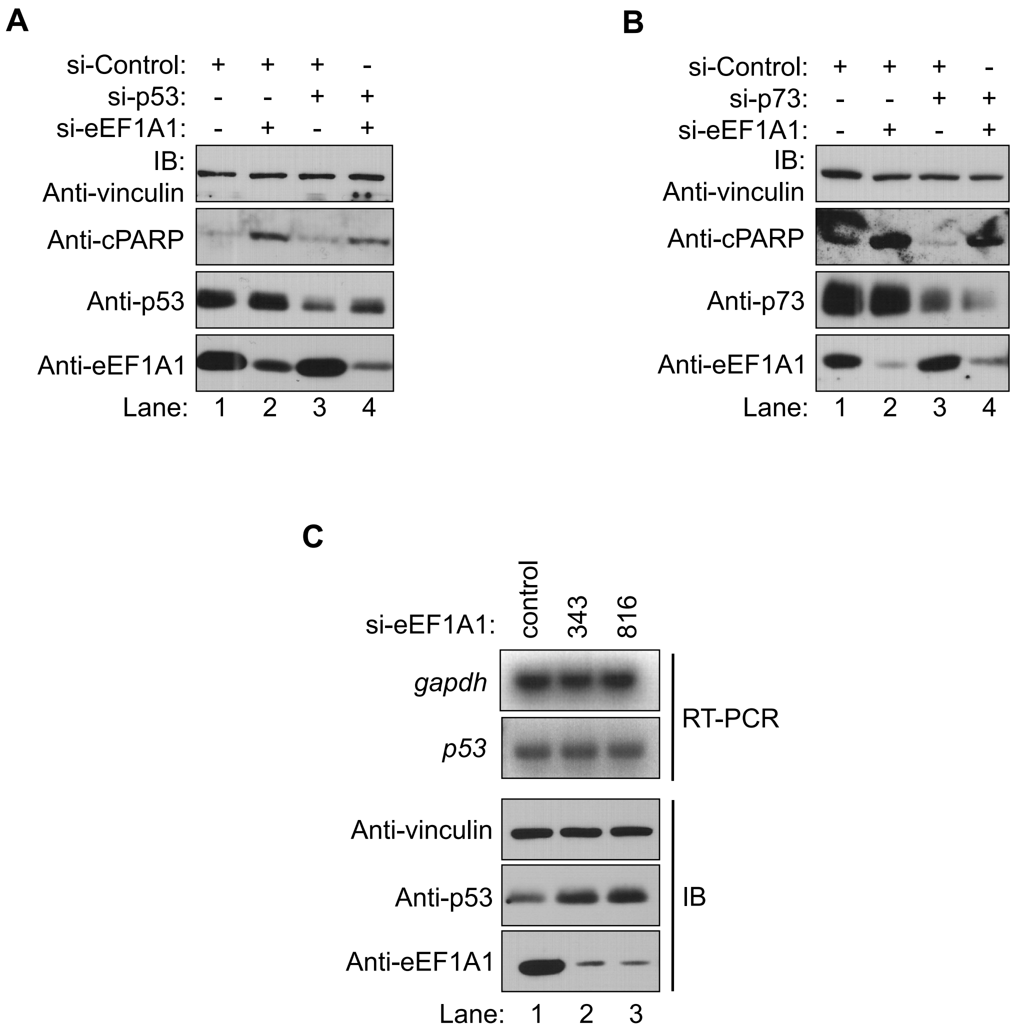

Figure S4. eEF1A1 is a negative regulator of p53 and p73 dependent apoptosis. HEK293 cells were transfected with siRNA oligonucleotides specific for eEF1A1 and/or p53 (panel A) or p73 (panel B), and treated with cisplatin (2 μM) for 18 hours. Whole cell extracts were resolved by SDS-PAGE and immunoblotted with the indicated antibodies. **Figure S4C,** HeLa cells were transfected with two different siRNA oligonucleotides specific for eEF1A1 or control. RNA was isolated and subjected to RT-PCR using the indicated primers. A fraction of cells were lysed and whole cell extracts were immunoblotted with the indicated antibodies.
